# Exploring How Professionals Within Agile Health Care Informatics Perceive Visualizations of Log File Analyses: Observational Study Followed by a Focus Group Interview

**DOI:** 10.2196/14424

**Published:** 2020-01-21

**Authors:** Iris ten Klooster, Matthijs Leendert Noordzij, Saskia Marion Kelders

**Affiliations:** 1 University of Twente Faculty of Behavioral, Management, and Social Sciences Department of Psychology, Health, and Technology Enschede Netherlands; 2 Saxion University of Applied Sciences Department of Psychology and Human Resource Management Deventer Netherlands; 3 North West University Optentia Research Focus Area Vanderbijpark South Africa

**Keywords:** log file analyses, user-centered design, agile, Markov Chains, health care systems

## Abstract

**Background:**

An increasing number of software companies work according to the agile software development method, which is difficult to integrate with user-centered design (UCD) practices. Log file analysis may provide opportunities for integrating UCD practices in the agile process. However, research within health care information technology mostly has a theoretical approach and is often focused on the researcher’s interpretation of log file analyses.

**Objective:**

We aimed to propose a systematic approach to log file analysis in this study and present this to developers to explore how they react and interpret this approach in the context of a real-world health care information system, in an attempt to answer the following question: How may log file analyses contribute to increasing the match between the health care system and its users, within the agile development method, according to agile team members?

**Methods:**

This study comprised 2 phases to answer the research question. In the first phase, log files were collected from a health care information system and subsequently analyzed (summarizing sequential patterns, heat mapping, and clustering). In the second phase, the results of these analyses are presented to agile professionals during a focus group interview. The interpretations of the agile professionals are analyzed by open axial coding.

**Results:**

Log file data of 17,924 user sessions and, in total, 176,678 activities were collected. We found that the Patient Timeline is mainly visited, with 23,707 (23,707/176,678; 13.42%) visits in total. The main unique user session occurred in 5.99% (1074/17,924) of all user sessions, and this comprised Insert Measurement Values for Patient and Patient Timeline, followed by the page Patient Settings and, finally, Patient Treatment Plan. In the heat map, we found that users often navigated to the pages Insert Measurement Values and Load Messages Collaborate. Finally, in the cluster analysis, we found 5 clusters, namely, the Information-seeking cluster, the Collaborative cluster, the Mixed cluster, the Administrative cluster, and the Patient-oriented cluster. We found that the interpretations of these results by agile professionals are related to stating hypotheses (n=34), comparing paths (n=31), benchmarking (n=22), and prioritizing (n=17).

**Conclusions:**

We found that analyzing log files provides agile professionals valuable insights into users’ behavior. Therefore, we argue that log file analyses should be used within agile development to inform professionals about users’ behavior. In this way, further UCD research can be informed by these results, making the methods less labor intensive. Moreover, we argue that these translations to an approach for further UCD research will be carried out by UCD specialists, as they are able to infer which goals the user had when going through these paths when looking at the log data.

## Introduction

### Background

User-centered design (UCD) is a vital determinant of health care informatics’ success, as it leads to quality improvement, resource savings, increased user satisfaction, and, ultimately, better patient care [1,2]. However, an increasing number of software companies work according to the agile software development method (agile), which is difficult to integrate with UCD practices [3]. Agile is defined in the Oxford Dictionary as “Able to move quickly and easily,” and it also refers to multiple methods within software development, which act as a counterpart to the previously used waterfall method. Examples of these agile methods within software development are Scrum, Extreme Programming, and dynamic systems development method. Waterfall methods follow predefined steps in a fixed order, whereas agile is characterized by its small software releases, with rapid iterations of 2 to 4 weeks. Every iteration can be seen as a small project in itself, and after every iteration, the product is shown and tested, and the product and process are evaluated [4]. Two explanatory factors are regularly mentioned in the literature as a cause of the difficult integration of agile and UCD.

The first one is that agile focuses on customer input instead of user involvement [3,5,6]. In the study by Gulliksen et al [6], it is explained that while working with agile, hardly any distinction is made between the customer and end user, raising the question whether the variation in user groups is overlooked. Moreover, the question may be asked as to how the needs of the customer, the one who pays for the health information technology, must be prioritized, given the presumable differences with users’ needs.

The second explanatory factor is that, although a resemblance between agile and UCD is the incorporation of iterations, there are large differences in the interpretation of these distinct iterations [7]. To begin with, agile focuses on testing code effectiveness during very short iterations, adding small pieces of very high-fidelity pieces to the health information technology. In UCD practices, we see that a Big Design Upfront approach is often used, using low-fidelity prototypes to communicate large pieces of the health information technology. When these UCD practices are fitted into agile, the costs for developing health care information technology strongly increase; therefore, the integration of UCD within agile is problematic and mostly lacking.

Log file analysis may provide opportunities for integrating UCD practices in the agile process. Log files give an objective view of users’ behavior in software systems and provide valuable insights in how health care information technology is used [8]. Gaining insight into users’ behavior may give indirect clues that are relevant to the objective of UCD, without actually having to involve the user in all the phases of development. Thus, the user perspective is added within the agile process, without the need to apply labor-intensive test methods. However, to our knowledge, no studies have been reported on if and how log file analyses can be used in practice within this agile development process. Therefore, we propose a systematic approach to log file analysis (including preprocessing, analysis, and various visualizations) in this study and present this to developers to explore how they react and interpret this approach. An important question is whether their perception of this information has the potential to increase the focus on the intended user within an agile development process.

**Figure 1:**
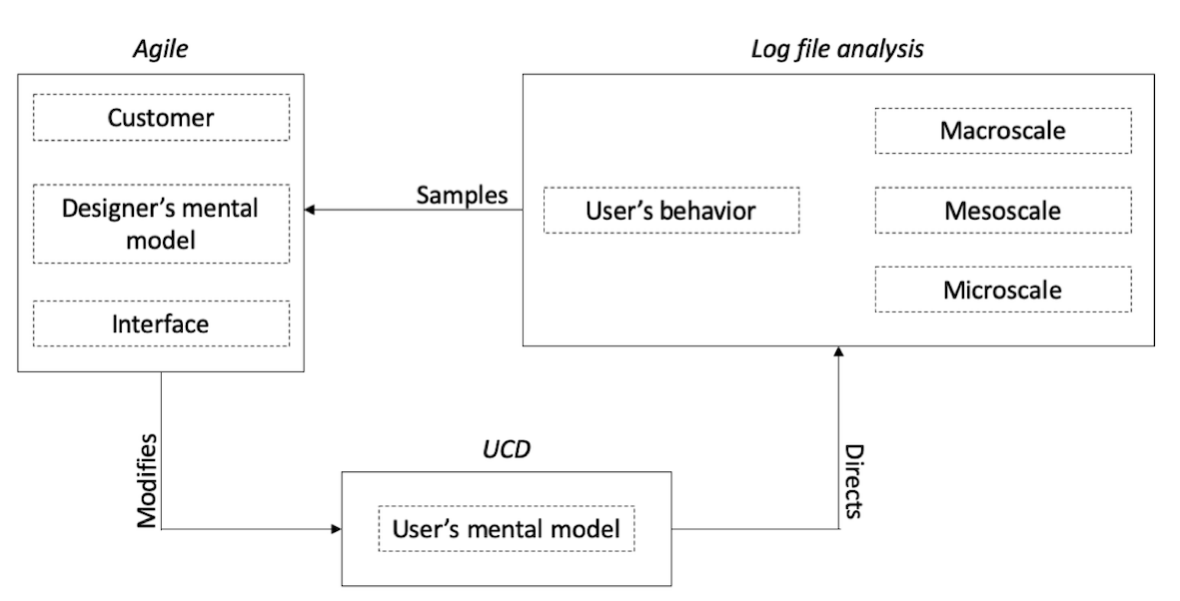
Graphical representation of the focus of the agile method (customer input and effective coding), user-centered design (mental models of the user), and log file analysis. This study focuses on the user’s behavior on the macroscale. UCD: user-centered design.

To further substantiate our expectations regarding the added value of log file analyses in the agile process, we will elaborate on the elements described above. In Figure 1, these elements and their relations are summarized. Here, we see that UCD practices in developing software systems means that end users have an influence in the development throughout the design process. Thus, the focus is on the users’ mental model of the system, comprising their expectations of how the system works. Within agile, the customer influences the development of software systems throughout the design process, which consequently influences the designer’s mental model, thereby guiding his development of the software system. This system then modifies the user’s mental model. In the following sections, we first introduce the reader to the relation between log file analysis and users’ mental models. We then describe an approach to log file analysis, which fits with describing users’ behavior on the macroscale. Finally, the aim of this study is described.

### Log Files and Users’ Mental Models

Log files are an automatically produced documentation of users’ behavior within a particular system [9]. These files provide information about the time of the event, the URL visited, and either posting or retrieving information from the system. Log files provide insight into the users’ real-life complex behavior while they are in their natural environment, and they are based on passive, unobtrusive monitoring. These characteristics are desirable in the increasingly complex world of health care informatics, and they compare favorably with laboratory testing, which is dependent on active, obtrusive monitoring and simplified, unengaging, and artificial use case scenarios [10]. Furthermore, log files can provide detailed information about individual users (intraindividual patterns), allowing for valid generalizations about patterns that might hold for groups of people (interindividual data) [11].

Users’ behavior is strongly impacted by their mental model of the technical system [12]. These mental models are the internal representations of the system, on which users make inferences on how to carry out tasks [12]. These tasks are carried out within the target system, on which an interface is created, allowing the user to interact with the system. When working according to a user-centered approach, agile team members try to fit the software to the users’ mental models. However, developers’ mental models mainly comprise how the system works, whereas users’ mental models comprise beliefs on how to use the system, leading to software that does not regularly fit with the mental models of the user [13]. A way to prevent this difference between the developers’ and users’ mental models is to get users in front of the interface and employ research methods to see whether this mental model is different from the developers’ mental model [13].

These research methods can be divided into several dimensions to gain insight into the characteristics and added value of these methods. In the study by Barkhuus and Rode [14], it is suggested to classify these methods as empiric or analytic and as quantitative or qualitative. A useful addition to this is the classification of methods on different time scales. On the basis of Newell’s bands of behavior [15], earlier research explored how insights into users’ behavior differ on the micro- (second-to-second), meso- (min-to-hours), and macroscale (week-to-month) [16]. To illustrate this, observing users’ behavior on the microscale provides insights into usability issues, mostly in lab settings, thereby allowing to collect data about the participants and control variables while they interact with the system. Another approach is observing users’ behavior on the mesoscale, in which data are collected on the affective responses to the system in a more natural setting. The most natural approach is collecting users’ data on the macroscale, providing an objective insight into the entire range of users’ behavior over long periods of time [16]. Given the advantages of analyzing users’ behavior on the macroscale, and the small usage of analytical quantitative methods in previous research [14], we used an analytical quantitative approach on the macroscale in this study.

### Log File Analysis

A method that fits well with describing users’ behavior on the macroscale is analyzing users’ behavior as a sequence of events [17]. This way of analyzing provides insight into the order in which the user navigates the system, whereas the more classical approach to log file analysis for health care information systems only describes quantities of usage (eg, number of times pages are visited, mean duration of visiting a page). In this analysis, there are some key concepts that play a role. First, a user session is a set of page views between logging into the system and logging out of the system, for a particular user at 1 particular website. Second, page categories are the distinct URLs in the log files grouped into categories.

#### Summarizing Sequential Patterns

Summarizing sequential patterns can be done by obtaining insight into the number of times that page categories and transitions between these page categories appear within users’ sessions. Moreover, it describes how many times page categories appear at a specific step within the user session. For example, the number of times that a specific page is visited as a first step within all users’ sessions. In earlier research related to health care information technology, these analyses provided insight into the efficiency of users navigating through the system [18].

#### Heat Mapping

Heat mapping provides functionalities to predict which page category a user will navigate to when he or she is visiting a specific page category. The probabilities of navigating from a specific page category to the other page categories are calculated, and these probabilities are shown on a heat map. The probabilities are calculated by means of Markov Chain modeling, meaning that, in contrast to summarizing sequential patterns, the purpose is to predict future usage behavior. Probabilities can be calculated by using zero-, first-, and higher-order Markov Chains [17]. These orders differ in that the next page category is predicted only on the basis of the current page category that is visited by the user (first order) or on a combination of the current page category with the page categories that the user was visiting before the current page category (higher order). A comparable approach was deployed in the study by Elizabeth and Cimino [19], where the insights were used to find clinicians’ information needs, which can be used to improve the design.

#### Clustering

In complex health care systems, it can be assumed that the users are heterogeneous, meaning that there is variation in their behavior [17]. Therefore, these user sessions can be clustered on the basis of the transitions between page categories. With clustering, the user behavior can be described in a global way, meaning that the focus is not on transitions between the distinct page categories. Instead, it focuses on all transitions between page categories, and subsequently, complete user sessions can be typified by their cluster name on the basis of the similarity of these user sessions. In the study by Vest and Jasperson [20], it was concluded that clustering provides a more thorough understanding of variation in users’ behavior, thus in the individual patterns that emerge.

### Aim of the Study

Overall, we see that analyses of users’ behavior through log files is used in several domains and that a small number of studies extended analyses related to quantities of usage with, for example, cluster analyses or analyzing sequential patterns. Moreover, research within health care information technology is focused on the researcher’s interpretation of these log file analyses, or a theoretical approach is used, in which log file data are correlated with other demographic data, so that the concrete approach toward improving health information technology development is not suggested. Finally, to our knowledge, no studies have been reported on the agile professionals’ interpretation of log file analyses; therefore, an opening to a coupling of these interpretations with concrete steps in the agile process is missing.

Therefore, the aforementioned 3 log file analyses were assessed in the context of a real-world health care information system, in an attempt to answer the following question: How may log file analyses contribute to increasing the match between the health care system and its users within the agile development method, according to agile team members. First, these log files were summarized, heat mapped, and clustered, respectively, to illustrate how users’ behavior can be described on the macroscale. Second, this study explored through a focus group interview whether and how agile team members can use these results within the agile software development method.

## Methods

### Phase 1: Collection and Analysis of the Log Files

Before the first phase of this study was carried out, permission was obtained from the Ethics Committee of the University of Twente, regarding collecting log files and analyzing these log files. The log files were treated as confidential and only kept on secured self-owned servers in Enschede, the Netherlands.

#### Data Collection

The log files were collected through an internet-based support system that serves as an extension of the general practitioner information system (GPIS). For privacy reasons, this system is appointed by the fictitious name Extendia. It provides general practitioners (GPs) with a comprehensive range of conceptually distinct services. It is a closed system, meaning that users are required to purchase a subscription before they can use it. When users directly log in via the browser, he or she will start within the Declarations area. When a user logs in via another connected system, he or she does not have to log in, and he or she starts on a different page, depending on the link the user has clicked on. Through the menu, users can download TeamViewer, open manuals, and navigate to their own profile. Depending on which parts they purchased, users can use the services Collaborate, Patients, Declarations, Dashboards, and Practice Web. They can navigate to these services via a main menu at the top. Within these services, users can navigate to the related subfunctions. Below, we briefly describe these distinct services.

1. Collaborate: Within collaborate, users can consult another specialist to ask a question about a patient. Second, users can refer a patient to a mental health group, while keeping the opportunity to gain insight in the treatment of that patient.
2. Patients: The patient area is strongly connected to the collaborate area, and it offers users the opportunity to gain insight in patients’ historical and current medication, as well as their measurement values. Moreover, historical care activities of the patient are clearly displayed in chronological order on the patient timeline. 
3. Declarations: The declarations area includes 4 services for doing declarations between different health care providers and insurers. Within this area, users do declarations, and these subsequently appear in the declaration overview. Next to this, the attention page gives an overview of rejected declarations. On the page Manual Invoices, users find and print (to send or provide) all patient invoices that are created in the health care systems’ mutation screen. Finally, there is an option for creating passant invoices and third-party invoices. 
4. Dashboards: The dashboard area offers users the opportunity for GPs to benchmark their results. They can make reports for insurance companies and reflect their results in comparison with colleagues on a financial level, as well as on practice and patient data.
5. Practice Web: This part of the system offers functionalities to the users to support daily practice. The main parts are an address book, a to-do list, and messages. In the address book, contact details of the institutions and care providers who are often approached with regard to the care surrounding the patients or the practice can be saved and be seen by all employees. Second, with the to-do list, actions can be assigned to the various employees, and users can keep track of who carried out the tasks and when these tasks were carried out. Finally, through the message component, internal communication is digitally recorded.

Log files were collected through this system at server side from September 18, 2017, to October 17, 2017. They were chosen for a set of data, covering a period of 1-month, to fit well in agile (with a maximum duration of sprints of 4 weeks).

#### Data Preparation

**Figure 2:**
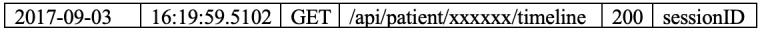
Example of a line in the collected log data.

There were 17,924 user sessions obtained from the health care system. These were cleaned before user sessions were analyzed, following the process described below. An example line of the log files collected at server side can be seen in Figure 2. This line of data comprises the date, the time, the method, the URL requested, the status code, and the session identification number. In this specific example, the user requests the timeline within the patient area for a specific patient on September 3, at around a quarter past 4.

The columns date, time, URL, and session id were used. To ensure privacy of the users, data were anonymized while keeping the opportunity to distinguish between sessions. Second, the URL variables were separated to allow for editing these variables separately, in subsequent steps of cleaning the data. Background processes were removed, to ensure that log data only comprised page views initiated by the users. Moreover, when users visit a page for a specific patient, a unique page identifier is inserted. To analyze these pages for all users together, these identifiers were removed. At last, date and time were combined into 1 variable to use these data in detail.

**Figure 3:**
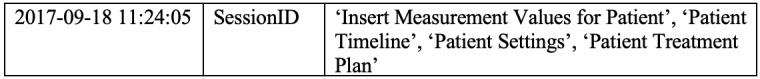
Cleaned data comprising comma-separated strings representing user sessions.

As a subsequent step, each URL was precategorized into 62 page categories (eg, Upload Patient File, Patient Timeline, and Address Book) and grouped by session numbers ordered by the variables date and time. This way, data of user sessions are formed. This is a set of views in a user session for a particular website [21], between logging into the system and logging out of the system. The server session ends when there is user inactivity for at least 30 min. The cleaned data comprise comma-separated strings representing user sessions. An example can be seen in Figure 3.

#### Data Analysis

Descriptive statistics were calculated, and a process map was obtained in Disco version 2.2.0 (Fluxicon) [22]. Subsequently, the log files were analyzed using the statistical software R (R Core Team) [23], within RStudio, version 1.0.143 (RStudio, Inc). Descriptive statistics were plotted using the ggplot2 package version 3.0.0, developed by Wickham [24]; a heat map was made, and log files were clustered using the Clickstream package version 1.3.0, developed by Scholz [17]. The number of clusters was chosen on the basis of an “elbow test.” These clusters were then typified on the basis of the pages that mainly occur within user sessions that fall within the distinct clusters.

### Phase 2: Focus Group and Qualitative Analysis

To explore how agile team members can use a description of users’ behavior on the macroscale obtained through log file analyses, a focus group interview was carried out. A focus group interview lends itself to questioning people in a more natural conversation pattern, so that it closely resembles a setting, as it occurs daily within agile.

#### Participants

The participants were obtained through purposive sampling. An invitation was sent via a digital calendar to all agile team members who worked at the collaboration department. In total, 10 Dutch-speaking employees worked at this department, of whom 7 participated in this study. Their mean age was 31.14 years (SD 4.88 years, range: 5-39 years). A total of 6 participants were male and 1 participant was female. Moreover, 3 participants were developers, 2 were designers, and 2 were information analysts.

#### Materials and Procedure

The focus group interview was carried out in an office within the software company. At the beginning of the focus group interview, the participants were asked whether they agreed with recording the interview, and they were told that these data would be processed anonymously. All the participants agreed with this; thereafter, the recording of the focus group interview was started. Notes were made during the interview, which served as a backup in case the recording was not usable.

Subsequently, the researcher introduced the focus group interview, and results of the log file analyses were shown. In the introduction, the participants were told that the results of the log file analyses were going to be presented and that the focus was on increasing the match between the system and its users and not on the performance of the system. A number of examples were given, and they were told in which period the data were collected. To increase their input, they were told that they could respond to the analyses that they were going to see and that there were no right or wrong responses. After this introduction, the results were presented on a beamer in the following order: frequency overview of pages developed by the collaboration department, heat maps (only pages of the collaboration department included), and the process map of all pages. The clusters were not used during the interview, as this would make it necessary to include results related to parts developed by other departments. The focus group interview continued until there was no more input from the participants. This way, it was decided that sufficient data had been collected.

#### Analysis

Before the analysis, the focus group interview was literally typed out using the F5 program. These transcriptions were imported into Atlas.ti version 8.1.3 to analyze the qualitative data. The data analysis took place by means of the coding method described in the study by Onwuegbuzie et al [25]: constant comparison analysis. The statements made by participants were evaluated by interpreting the meaning and assigning it a value code (open coding). A code was assigned to relevant information per fragment. (Parts of) Fragments received a maximum of 1 code. These codes were then grouped into overarching codes. Thereafter, links were sought among the concepts, associations, and combinations. This resulted in main groups and subgroups (axial coding). A total of 10% of the data was double coded: once by the researcher and once by a colleague of the researcher. The interrater reliability was calculated by means of Cohen kappa in SPSS version 23, and it was found to be acceptable at 0.81.

## Results

### Phase 1: Collection and Analysis of the Log Files

To illustrate analyses that were shown to the agile team members, we describe the results of the first phase of this study. In this phase of the study, log files that were collected on server side were analyzed.

#### General Results

In total, 176,678 activities were conducted within 17,924 user sessions, an average of 9.86 activities per user session. In Multimedia Appendix 1, an overview of the distribution of these user sessions over the days is given. On weekdays, the number of user sessions is higher (mean 776.14) than during weekend days (mean 96.38).

The page Patient timeline was the most visited page within these user sessions (n=23,707), on which historical care activities of the patient are displayed in chronological order. In addition, the page Insert Measurement Values was then the most visited page (n=21,215). Table 1 shows the frequency and percentage of visits of the 6 pages that were visited most.

**Table 1:** Most visited pages within the health care system between September 18, 2017, and October 17, 2017 (N=176,678).

Page	Times visited, n (%)
Patient Timeline	23,707 (13.42)
Insert Measurement Values for Patient	21,215 (12.01)
Patient Settings	20,306 (11.49)
Load Messages Collaborate	17,284 (9.78)
View Report	15,472 (8.76)
Patient Treatment Plan	14,814 (8.38)

The least visited page was Change conversation topic (n=3). This page within Collaborate is used for conversations among several health care providers. Here, the various care providers can consult each other about the care they give to a patient. Changing the subject of these conversations was done least within the health care system. Table 2 shows the frequency and percentage of visits of the 5 pages that were least visited.

**Table 2:** Least visited pages within the health care system between September 18, 2017, and October 17, 2017 (N=176,678).

Page	Times visited, n (%)
Add Participants	20 (0.01)
Insight Number References to Mental Health Group	17 (0.01)
To-Do List	10 (0.01)
Delete Participants	7 (0.00)
Change Conversation Topic	3 (0.00)

#### Summarizing Sequential Patterns

**Figure 4:**
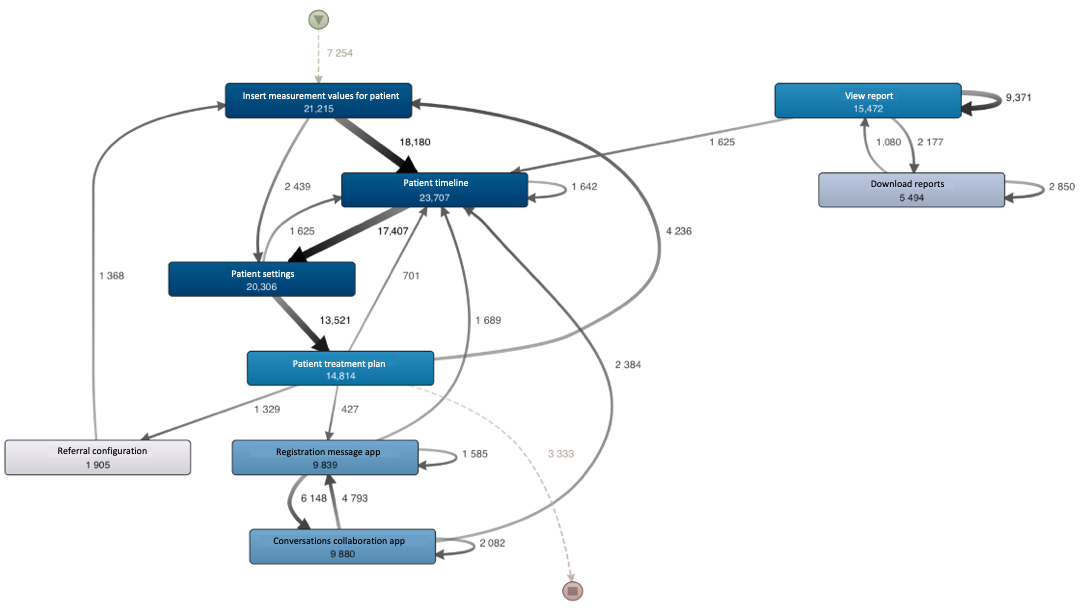
Overview of how users generally navigate through the system. The start of the process is illustrated by the triangle symbol at the top of the process map. Similarly, the end of the process is illustrated by the stop symbol. Pages are represented by boxes and the process flow between 2 pages is visualized by an arrow. Dashed arrows point to page visits that occurred at the very beginning or at the very end of the process. The absolute frequencies are displayed in the numbers at the arcs and in the boxes. The thickness of the arrows and the coloring of the activities visually support these numbers.

In total, 6111 unique user sessions were found within all 17,924 user sessions. Overall, there is a lot of variation in how users navigate through the system. This section focuses on the paths that generally occur within user sessions. To begin with, the main unique user session occurred in 5.99% (1074/17,924) of all user sessions; this comprised Insert Measurement Values for Patient and Patient Timeline, followed by the page Patient Settings and, finally, Patient Treatment Plan. To gain an understanding of the sequence of all user sessions, a process map was obtained in Fluxicon Disco. This process map can be seen in Figure 4, in which the level of detail for Activities (or page categories) was set to 8.8% and Paths (or transition between pages) was set to 3.7% in Fluxicon Disco.

In Figure 4, we can see that there are 7254 user sessions in the dataset, which all start with the page Insert Measurement Values for Patient. Moreover, users navigated to the page Insert Measurement Values for Patient from the pages Patient Treatment Plan (n=4236) and Referral Configuration (n=1368). Thereafter, the user sessions split into 2 alternative paths: In 18,180 user sessions, the page Patient Timeline was visited after Patient Treatment Plan. The other 2439 user sessions navigated to Patient Settings instead. In total, the page Insert Measurement Values was visited most often (in total 21,215 times), which is more than the number of user sessions within the data (n=17,924). This comes from the dominant loop that goes indirectly through the Patient Timeline. Repeatedly, the Patient Treatment Plan was reanalyzed while observing and inserting measurement values for the patient.

Another remarkable result in this figure is how and where the pages View Report and Download Reports occur in the user sessions, as they are graphically far removed from the other page categories. Moreover, there is an arrow pointing from and toward both categories, indicating repetition in visiting these page categories. The page View Report was visited 15,472 times, which allows users to make reports for insurance companies and reflects their results in comparison to colleagues on a financial level, as well as on practice and patient data. From this page, 2177 users continued their path with visiting Download Reports. This page (Download Reports) was visited 5494 times, from which 1080 users visited View Report. Similarly, after downloading reports within the health care system, 2850 users continued their path with downloading reports. There are a number of such patterns in the process map. For the readability of this study, these are not all mentioned in the text (see Figure 4 for patterns). Figure 4 also shows that, generally, user sessions ended with visiting the page Patient Treatment Plan.

#### Heat Mapping Log Files

**Figure 5:**
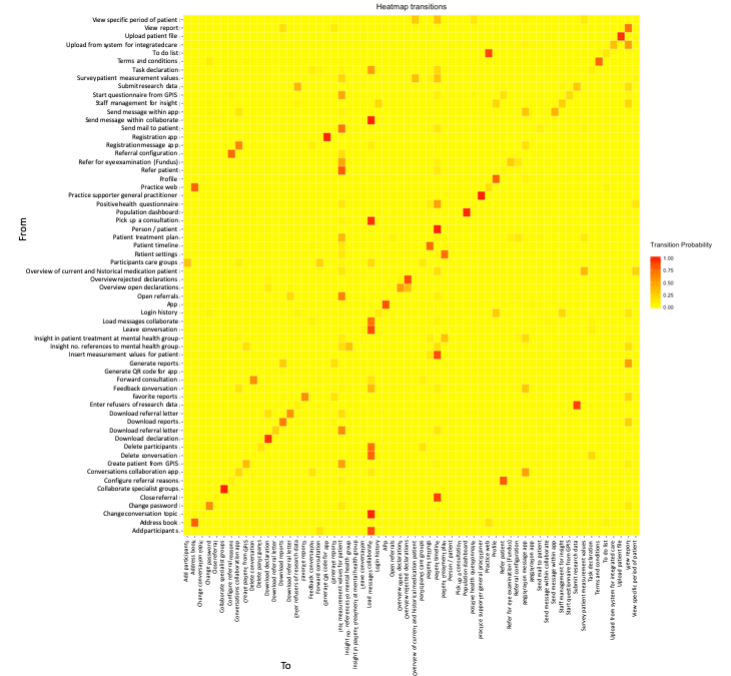
Heat map of log files. The pages on the vertical line show from which page the user navigates, and the horizontal line shows the pages toward which page a user navigates, where yellow squares stand for low transition probabilities, red squares stand for high transition probabilities, and orange squares stand for intermediate transition probabilities. GPIS: general practitioner information system.

To explore how users’ behavior can be described by a heat map, log files were modeled with a first-order Markov Chain. A heat map was plotted for this transition matrix, with the y-axis representing the current page category and the x-axis representing the next page category. In Figure 5, we see that users often navigated to the pages Insert Measurement Values and Load Messages Collaborate. Moreover, we see that there is a high probability of users repeatedly navigating from page Address Book toward the page category Address Book again. 

This page was visited for a total of 133 times in a 1-month period. A page visited for a small number of times such as this, relatively, would not appear quickly in a process map that gives an overview of the main visited pages.

#### Clustering User Sessions

Finally, the user sessions were divided into clusters with similar browsing patterns (see Table 3). First, 1 randomly chosen day in the log files was picked for the cluster analysis because of limited computational power. These data were then divided into 5 clusters, on the basis of the “elbow test.” The clusters were typified on the basis of the pages that appear in these clusters. The 5 clusters are named as the Information-seeking cluster (sum of squares [SS]=96.16), the Collaborative cluster (SS=99.27), the Mixed cluster (SS=193.40), the Administrative cluster (177.57), and the Patient-Oriented cluster (SS=378.02). The total SS within groups was 944.42 and the between SS was 561.49.

Cluster 1, the Mixed cluster (n=311), shows a clear variance of events from event categories View Report, Registration Message Application, and Conversations Application Collaborate. As most user sessions belong to this cluster, these can be interpreted to be the most typical sessions. This shows that the most typical user session involves a variety of tasks that can be performed within the system, meaning that users value the wide range of tasks offered by the system.

Cluster 2, the Patient-oriented cluster (n=277), is also a very dominant cluster, as it is the second most common among the user sessions. User sessions that fall within this cluster are oriented around the patient. Users are actively searching for a patient’s timeline, changing the settings of the patient, and entering measurements for that patient. This cluster seems to coincide with the layout of the system, of which a part is divided into searching and inserting patient data.

**Table 3:** Clusters with similar browsing patterns, along with the number of user sessions that were grouped into these clusters and the 3 most visited pages within the clusters.

Cluster	User sessions within cluster, n	Most visited page	Second most visited page	Third most visited page
Mixed	311	View Report	Registration Message Application	Conversations Application Collaborate
Patient-oriented	277	Patient Timeline	Patient Settings	Insert Measurement Values for Patient
Information-seeking	64	Download Reports	View Report	Population Dashboard
Collaborative	54	Load Messages Collaborate	Registration Message Application	Conversations Application Collaborate
Administrative	15	Download Declaration	Overview Rejected Declarations	Download Referral Letter

Cluster 3 is the Information-seeking cluster (n=64). This cluster shows a dominance of page categories Download reports, View Report, and Population Dashboard. It is the most information behavior–related cluster, as it reveals the users searching for which patients have visited the GP and what they have done there through reports.

The Collaborative cluster (n=54) shows a dominance of sessions involving collaboration with other care providers. The pattern of loading messages within Collaborate, followed by doing registrations for the message app and visiting this message app, shapes a picture of users looking at their notifications from other care providers and being actively involved in contacting these other providers.

Finally, the Administrative cluster (n=15) is the pattern that is least dominant in the user sessions. This cluster is typified by the administrative purposes that are performed within the sessions. The page categories are related to downloading declarations, looking at an overview of rejected declarations, and downloading referral letters.

### Phase 2: Focus Group and Qualitative Analysis

A total of 4 main codes were found in the qualitative data, namely, Hypotheses, Path comparison, Benchmarking, and Prioritize. The number of times codes were found in the data can be found in Table 4. In this section, these codes will be defined, and the variance will be described using prime examples.

**Table 4:** Main categories and subcategories found in the qualitative data.

Main category and subcategory		Count (n)
**Hypotheses**		34
	Usability problems	19
	User requirements	6
	Other systems	10
**Path comparison**		31
	Logical paths	13
	Illogical paths	12
	Incomplete paths	4
**Benchmarking**		22
	Page visits	19
	Users	3
**Prioritize**		17
	Redesign	4
	Making improvements	13

#### Hypotheses

The code Hypotheses was found 34 times in the data obtained through the focus group interview. On the basis of the results of the quantitative phase of this study, participants mainly communicated ideas about the cause of these results. These hypotheses of the participants were related to usability problems, user requirements, or the way of working in combination with other systems. Usability problems were often mentioned while discussing the heat maps. Participants focused mainly on the diagonal line of the heat maps to think of usability problems. On this line, participants could see how often a specific page is followed by loading the same page again. Examples of usability problems that were mentioned are lack of feedback, lack of clarity as the system is often adjusted, or entering incorrect passwords. An example of a usability problem related to the lack of feedback was mentioned by participant 1:

Yes, we have just made a disable for that. Because Teledia received double or triple registrations. You had to press “sign up” there and then you had to wait. But there was nothing to indicate that it was logging in at that time. That’s why they clicked again: they thought “nothing happens.”

Participant 1, information analyst

Participants also formulated hypotheses that were related to user requirements related to functions that are unavailable within the system, on the basis of the heat maps and frequencies of page visits. For example, respondents mentioned that deleting messages occurred much more often than leaving the conversation. Moreover, the heat map revealed that after opening a conversation, users are inclined to directly delete the message. When a message is deleted, users cannot obtain the information about these messages anymore, whereas when leaving a conversation, this possibility is retained. Thus, according to the participants, there is no need for an archive function. An example of a participant mentioning this is the following:

Well, if you leave the conversation then you say: “Ok, I have nothing to do with this conversation, but I want to be able to read it later.”

Participant 1, information analyst

Finally, participants formulated hypotheses related to the way the system is used along with other systems, as well as reasons for this way of working with the other systems. Usability problems are also formulated on the basis of these hypotheses. Participants are focused on the page from which users go to another system or the other way around. The movement toward another system indicates missing parts in the system. Moreover, when users move toward the system from another system, this is an indication for functions that users find valuable. They inferred this because of the fact that users have to take extra steps from another system to use these functions. Moreover, based on the heat map, participants form hypotheses on the basis of the diagonal line. When there is a lot of repetition, users see another system as the starting point for carrying out their tasks. An example is the repetition of navigating to Extendia page categories from a GPIS. On the basis of this, the participants also formulate usability problems, for example, there is not a possibility to search on the basis of maiden name. Participant 6 mentioned this (this quotation is related to a loop that occurred in the process map):

The user is working from GPIS, and goes to Extendia via health portal with a button navigating to Extendia. When you do this a new tab opens with that patient information. And when you’re done, you close that tab again and then you go to your GPIS again, where the user searches the next patient. From there the user goes back to the Extendia button and then Extendia opens again.

Participant 1, information analyst

#### Path Comparison

The code Path comparison was found 31 times in the data obtained through the semistructured interview. On the basis of the results of the quantitative phase of this study, participants compared the paths that occurred in the log data with their expectations of how users navigate through the system. Paths are defined here as a sequence of requests to the server in time (as shown in the heat map and process map). The comparison of paths in the results with their expectations was logical, illogical, or incomplete. Participants mentioned logical paths through the system, by which they mention that users carry out the steps in the right order to fulfill their tasks. This gives reason to state that certain parts of the system must be maintained in the same way. An example of a participant mentioning this is the following:

But that is to be expected right. You start a conversation with someone. You get an answer, and then you’re done.

Participant 3, developer

Contrary to mentioning paths that match their expectations, participants also mentioned paths through the system, which did not match their expectations (process map and heat map). Moreover, they mentioned results indicating that users carry out certain steps toward fulfilling a certain task, but users do not complete these tasks (do not go through the final steps). An example of such an incomplete path was described as follows:

But now it becomes very complicated because the user does not close the referral. At a given moment this patient still appears to be in the overview. Then that user calls us to ask how this referral can be closed, and then we have to search for a long time, and then we finally come to the conclusion that the user has not closed the referral at the end.

Participant 3, developer

#### Benchmarking

Third, participants formulated thoughts related to the frequencies that were displayed, regarding the use of the system. They wanted to compare these frequencies with other frequencies to obtain valuable clues about possible navigation problems within the system. These comments were coded as Benchmarking, and these appeared 22 times in the qualitative data. First, the participants compared the number of page visits with another number of page visits to get an idea of the use relative to another function. For example, they compared the number of times the conversation topics were changed with the total number of messages that were opened. This gave indications for whether users find these functions valuable. Second, it seemed useful to the participants to compare frequencies of the use in the past with the current frequencies of use. For example, they could see whether recent developments concerning new users led to a logical increase in the number of page visits. An example of a participant mentioning this is the following:

So, if you analyze the frequencies, there is an opportunity to see whether the usage increases enough when a new care group is added to the system. If not, this gives reason to think something is not going well with the implementation of the system for the new users.

Participant 1, information analyst

#### Prioritize

Finally, participants indicated opportunities for the results to be used for prioritizing within the agile development process. This code, Prioritizing, occurred 17 times in the data. First, they mentioned some cues in the results for prioritizing a redesign of the system. To illustrate, if users had to navigate to a frequently visited page via a drop-down menu, this would give priority to redesigning this page. Navigating to this page should then be possible via a main menu. This was also the opposite for less visited pages. Second, they mentioned that the frequency of page visits provides clues for increasing or decreasing priority for making improvements to the system. An example of this is a participant mentioning the following:

Well, I think this is a very interesting result, because my colleague is doing a lot to improve that page. That is adjusted every time, and uhhhh, that kind of things, but this page is almost never used.

Participant 1, information analyst

## Discussion

### Principal Findings

The goal of this study was to explore the usefulness of log file analyses within the agile development method to increase the match between the health care system and its users. We have found that analyzing log files seems to provide agile professionals valuable insights into users’ behavior. This is in line with previous research related to health information technology, in which it was shown that researchers interpreted log file analyses into valuable insights into users’ behavior [8,18,26]. The important innovation of this study is that researchers looked at what you can do with these log file analyses and professional agile team members were also asked about how they can use these in practice. This is obviously very valuable, as health care information technology is mostly developed in software companies working according to agile. Below, we will elaborate on the contribution of our study.

First, we illustrated the results of summarizing sequential patterns, which provided insight into the prominent order that occurs within user sessions. In our view, the prominent order was related to the effectiveness with which users navigate through the system, which is in line with the research carried out in the study by Sieverink et al [18]. For example, the loop between View Report and Download Reports indicated that users repeatedly download reports after having viewed them. If the system would have offered an option to download several reports at once, these navigation paths could be more efficient. Agile team members were mainly interested in comparing expected paths with the actual paths in which users navigate through the system, while looking at summaries of sequential patterns.

Second, we described how, in our view, heat mapping log files provided insight into the transitions between lesser visited pages within the system. Thus, this was a more detailed overview of the simplified representation of summarizing sequential patterns. The addition of the heat map gave valuable prompts for possible bottlenecks in key functions within the system. It may be that a page is not visited often, but it also may be that it does have an important function. For example, the link between GPIS and the health care system only needs to be made once, but if this does not work, the health care system will be virtually unusable. The agile team members focused mainly on the diagonal line for stating hypotheses concerning usability problems. On this diagonal line, agile team members saw repetitions in page visits by the users. This approach is different from the one used in the study by Elizabeth and Cimino [19]. In that study, they tried to find out information needs on the basis of this sort of analyses, where we found that it mainly provides insight into possible usability problems. The reason for this is probably the different form of log data (search query logs), which was used in that study [19], as Web logs of search queries are only related to a specific part of the system. Moreover, analyzing search queries is not necessarily related to existing components within the system.

Third, the clusters provided insight into groups of user sessions led by similar mental models of the system, which is in line with the study by Huerta and McAlearney [26]. The variation that exists between user sessions could be summarized in 5 clusters, creating an understanding of differences in user behavior. In this way, the user behavior can also be looked at in a more personalized way. We also saw a clear example of how mental models can change during the use of a system. For example, the Administrative cluster was the least dominant cluster, whereas in the first instance, the system only offered tasks related to administration. The current approach of log file analysis differs from, for example, clustering user groups on the basis of survey and medical record data [27], as well as on the basis of interview data [28]. Although these approaches all aim for a description of user groups, the approach used in this study has the advantage of not requiring labor-intensive data collection that is often unfeasible within the agile process. However, the current approach can only be realized once (a part of) the system is already developed and available to its users. Therefore, one of the approaches, as described in the studies by Holden et al [27,28], is recommended when one is at the start of the health care informatics development process. When this initial stage of the development process has passed, the current approach might be used as a solution to the lacking distinction that is made between the customer and the user within agile [3,5,6]. By creating insight into the variation in user behavior, agile professionals are forced to see whether the input from customers and users within agile provides enough insight into users’ behavior, by comparing this with the full range of user behavior observed in the cluster analysis.

These findings give rise to recommending several ways in which log files analyses can be fitted into the agile process. To begin with, in the study by Russell [16], investigating user behavior at all 3 levels (micro, meso, and macroscale) was suggested so that a more complete picture is obtained to improve or evaluate the system. For example, usability problems can be found on the microscale, and this can be supplemented with affective responses found on the mesoscale to obtain a more complete view. However, in this study, it was found that on the basis of analyzing user behavior on the macroscale, hypotheses can be drawn about users’ behavior on the other scales. Therefore, we argue that not all scales should be described at the same time but that insight in macroscale users’ behavior should be used to adjust the methods for collecting data about users’ behavior on the other scales. To illustrate this, a hypothesis concerning usability problems related to the main menu, on the basis of log file analyses (macroscale), will then result in an interview with users about their response to this specific main menu (mesoscale). In the study by Gulliksen et al [6], it was claimed that the iterations of the agile process are too fast for adopting UCD methods properly, and by giving directions to these UCD methods with log file analyses, the time to collect data on specific parts can be largely shortened by only focusing on specific parts. We argue that these translations to an approach for further UCD research will be carried out by UCD specialists, as they are able to infer which goals the user had when going through these paths, while looking at the log data [29]. At the same time, analyses of macroscale users’ behavior enable the UCD specialists to be heard more in agile software development [6], as we found that agile professionals received the log file analyses positively during the interview.

### Limitations

The qualitative part of this study was based on an elaborate focus interview with relevant stakeholders. However, no follow-up or additional interviews were done to establish saturation with respect to the themes suggested here. It is important to note that the focus interview continued until all stakeholders felt they had contributed all they could, and in this way, it was established that enough data had been collected. Nevertheless, now that we have put forward a method to integrate the log files in the agile process, it would be of value to replicate our qualitative themes in a similar environment.

A further limitation is the generalizability of this study. The data of this study were collected at a large-scale software company developing health care software. However, no data were collected of small-scale (1 team) or very large-scale (more than 10 teams) software companies. Agile team members of small-scale and very large-scale software companies might have different views on ways in which insights into users’ behavior might be used because of the differences in coordination approaches during software development [30]. However, the goal of this study was to explore how a description of users’ behavior within a complex health care system can be of added value within the agile development method. Complex health care software systems are defined as systems that comprise several components, which must also be able to function independently. On the one hand, it can be assumed that the more complex health care systems are developed within larger software companies. On the other hand, the cluster analysis could be useful in small software companies, as they work with less separate departments on the software, allowing agile team members to understand the meaning of the clusters. Importantly, this study provides a framework on how to examine the usefulness of and provides a starting point for integrating log file information in agile development in a variety of contexts. To overcome the previously mentioned limitations, this framework can be used in follow-up research investigating the added value in small-scale and very large-scale software companies.

## Appendix

Multimedia Appendix 1 Number of user sessions over the days between September 18, 2017, and October 17, 2017.

## Abbreviations

GP: general practitioner
GPIS: general practitioner information system
SS: sum of squares
UCD: user-centered design
