# Supplementary figures and images for: Exploring How Professionals Within Agile Health Care Informatics Perceive Visualizations of Log File Analyses: Observational Study Followed by a Focus Group Interview
Source: JMIR Hum Factors. 2020 Jan 21;7(1):e14424. doi: 10.2196/14424 (PMC7001047; doi:10.2196/14424)

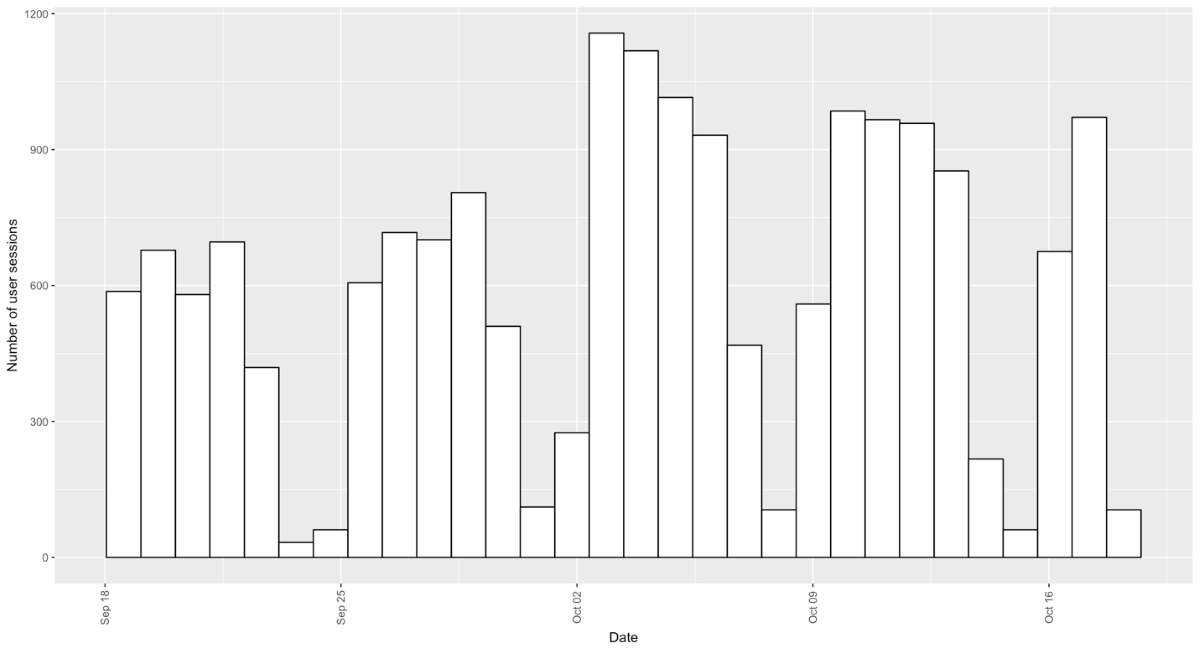

Supplement: Multimedia Appendix 1 [file humanfactors_v7i1e14424_app1.png]
